# A novel regulated network mediated by downregulation *HIF1A-AS2* lncRNA impairs placental angiogenesis by promoting ANGPTL4 expression in preeclampsia

**DOI:** 10.3389/fcell.2022.837000

**Published:** 2022-08-09

**Authors:** Lijun Shu, Cong Wang, Zhengzheng Ding, Jianjiao Tang, Yuanyuan Zhu, Liuxin Wu, Zheyue Wang, Tingting Zhang, Tianjun Wang, Yetao Xu, Lizhou Sun

**Affiliations:** ^1^ Department of Obstetrics and Gynecology, First Affiliated Hospital of Nanjing Medical University, Nanjing, JS, China; ^2^ Key Laboratory of Antibody Technique of National Health Commission, Nanjing Medical University, Nanjing, JS, China

**Keywords:** preeclampsia, lncRNA, HIF1A-AS2, placental angiogenesis, ANGPTL4, LNMA

## Abstract

Preeclampsia (PE) is the predominant medical condition leading to maternal and fetal mortality, and the lack of effective treatment increases its risk to the public health. Among the numerous predisposing factors, the ineffectual remodeling of the uterine spiral arteries, which can induce abnormal placental angiogenesis, has been focused to solve the pathogenesis of PE. According to the preceding research results, abnormal expression of long non-coding RNAs (lncRNA)s could be associated with the pathological changes inducing PE. To be more specific, lncRNA *HIF1A-AS2* was proposed for its potential to participate in the molecular mechanisms underlying PE. *In vitro*, in trophoblast cell lines HTR-8/SVneo and human umbilical vein endothelial cells HUVECs, *HIF1A-AS2* knockdown inhibited cell proliferation, migration and tube formation. Mechanistically, transcription factor FOXP1 could regulate the expression of *HIF1A-AS2*. Moreover, a series of assays, including RNA pull down and mass spectrometry, RNA immunoprecipitation and chromatin immunoprecipitation assay, revealed that *HIF1A-AS2* interacted with Lamin A/C (LMNA) to inhibit ANGPTL4 expression in trophoblast cells, thus further participating in the progression of PE. Taken together, these findings suggested that further analysis on *HIF1A-AS2* could contribute to the development of prospective therapeutic strategy for PE.

## Introduction

Preeclampsia (PE) is a hypertensive disorder specifically occurred in pregnancy, which is characterized by the new onset of increased blood pressure and proteinuria after 20 weeks of pregnancy in a previously normotensive woman. According to the recent epidemiological estimations, PE impacts 3%–8% of pregnancy women worldwide (Bouter and Duvekot, 2020) and causes serious hazard to the health of mothers and their fetuses. Moreover, it has been generally admitted that PE has remarkable causal relationship with maternal and perinatal mortality, especially in low-income and middle-income countries (Ives et al., 2020; Aukes et al., 2021). Globally, PE could result in more than 50,000 maternal deaths annually (Ananth et al., 2021). Concerning the limitations of the existing medical treatment, the conventional choice of drug for the alleviation of PE is aspirin, but it is unable to prevent the development of the disease. Although novel therapies for PE are being developed, placenta delivery is still the main and only effective treatment for PE (Mol et al., 2016).

Because PE originate in the placenta, placental dysfunction underlies a spectrum of PE-related symptoms (Phipps et al., 2019; Stepan et al., 2020). To induce PE, the aberrant development and dysfunction of placenta can be associated with abnormal remodeling of maternal uterine arteries, the main cause of which is impaired extravillous trophoblast (EVT) functional phenotype. Many papers proposed that inadequate EVT invasion could prevent the proper engrafting and remodeling of uterine spiral arteries, consequently inducing the occurrence of PE (Redline and Patterson, 1995; Staff, 2019; Staff et al., 2020). Overall, it is suggested to further explore the mechanisms underlying the abnormal EVT functional phenotypes and spiral artery remodeling, thereby expanding the theoretical basis for improving the clinical treatment of PE.

Long non-coding RNA (lncRNA) is defined by its length (more than 200 nucleotides) and the lack of protein-coding capability. The family of lncRNAs has been increasingly focused by various studies in recent years (Delas and Hannon, 2017; Palazzo and Koonin, 2020). To date, numerous lncRNAs have been recognized as critical factors to modulate biological functions, such as chromatin modifiers recruitment (Davidovich and Cech, 2015), immune response (Jiang et al., 2018), cell differentiation (Jiang et al., 2017), and disease-related dysfunctions (Wu et al., 2017; Hu et al., 2020). Aberrant expressions of lncRNAs are known to be closely related to various human diseases, as well as to the different stages of the diseases, such as the occurrence, the development, and the prevention, especially in PE (Xu et al., 2018a; Wu et al., 2018; Xu et al., 2020). For instance, the lncRNA *TUG1* modulates RND3 expression by binding to EZH2, in order to modulate cell proliferation, migration and network formation *in vitro* (Xu et al., 2017). Therefore, to improve the diagnosis and the treatment for PE, the key lncRNAs in the relevant molecular pathways should be identified, and their functions should be thoroughly investigated, which would contribute to the understanding of PE pathogenesis.

Hypoxia-inducible factor 1 alpha-antisense RNA 2 (*HIF1A-AS2*), a 2051-bp lncRNA gene, locates on chromosome14q23.2. Our previous study has reported that *HIF1A-AS2* expression in PE placental tissues were dramatically downregulated. *HIF1A-AS2* can participate in the invasive apoptotic biological process of trophoblast cells, in which it inhibits PHLDA1 expression by binding to LSD1 (Wu et al., 2019). Although lncRNAs can modulate the biological functions of cells through multiple mechanistic pathways, our particular interest is to explore how *HIF1A-AS2* participates in the pathways relating to angiogenesis. To reveal furthermore details of the relevant molecular pathways, this study was also designed to identify the upstream and downstream regulatory factors for *HIF1A-AS2* inducing the abnormal cellular activities in PE trophoblast cells, in order to recognize novel instructive clues for the improvement of clinical application in the future. Based on those findings, the association between PE and abnormal *HIF1A-AS2* expression was evaluated, in order to recognize novel instructive clues for the improvement of clinical application in the future.

## Results

### *HIF1A-AS2* promotes the proliferation, migration and invasion *in vitro*

**FIGURE 1:**
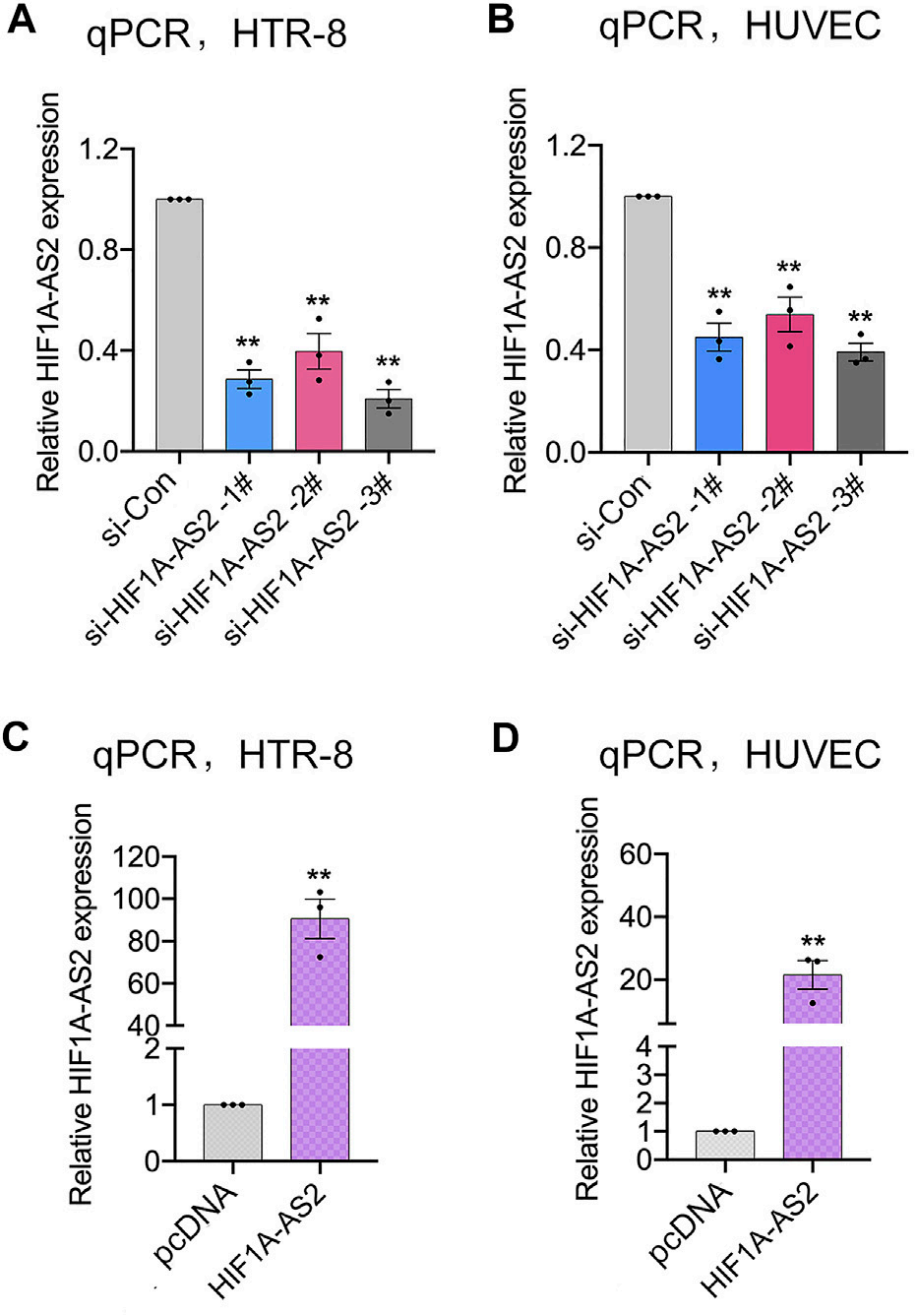
Modulation of *HIF1A-AS2* expression in trophoblasts. qPCR analysis of *HIF1A-AS2* expression in HTR-8/SVneo **(A)** and HUVECs **(B)** after transfected with *HIF1A-AS2*–specific siRNAs, respectively. *HIF1A-AS2* expression levels were tested by qPCR after treating with empty vector and pcDNA3.1-*HIF1A-AS2* plasmid in HTR-8/SVneo **(C)** and HUVECs **(D)**. Error bars are mean with SEM of technical replicates (*n* = 3). *p < 0.05, **p < 0.01; significance by Student’s t test.

Previously, our team demonstrated that *HIF1A-AS2* was dramatically down-expressed in PE placental tissue compared to the expression level of normal pregnant women. Meanwhile, the enhancement of *HIF1A-AS2* expression promoted the proliferation of trophoblast cells and thereby contribute to the uterine spiral artery recasting. The further analyses focused on the correlation between *HIF1A-AS2* expression and placental vasculature. The first step was to select trophoblast cell line (HTR-8/SVneo) and Human umbilical vein endothelial cells (HUVECs), which should be associated with placental angiogenesis and thus enable the demonstration of the relevant regulatory functions of *HIF1A-AS2*. To evaluate the influences of the changes in *HIF1A-AS2* expression on the placental blood vessels, *HIF1A-AS2* was silenced with specific siRNAs or overexpressed with plasmid (pcDNA3.1-*HIF1A-AS2*) in the two selected cell lines, respectively. To exclude off-target effects, three different siRNAs were specifically designed. After the transfection with siRNAs into the HTR-8/SVneo and HUVEC, qPCR assays confirmed that *HIF1A-AS2* expression was silenced in the cells treated with si-*HIF1A-AS2* 1#, si*HIF1A-AS2* 2# and si*HIF1A-AS2* 3#, respectively (Figures 1A,B). Therefore, si-*HIF1A-AS2*-1# and/or si-*HIF1A-AS2*-3# were used for subsequent experiments. Depending on the application of pcDNA3.1-*HIF1A-AS2* plasmid, the upregulation of *HIF1A-AS2* expression was also achieved (Figures 1C,D).

**FIGURE 2:**
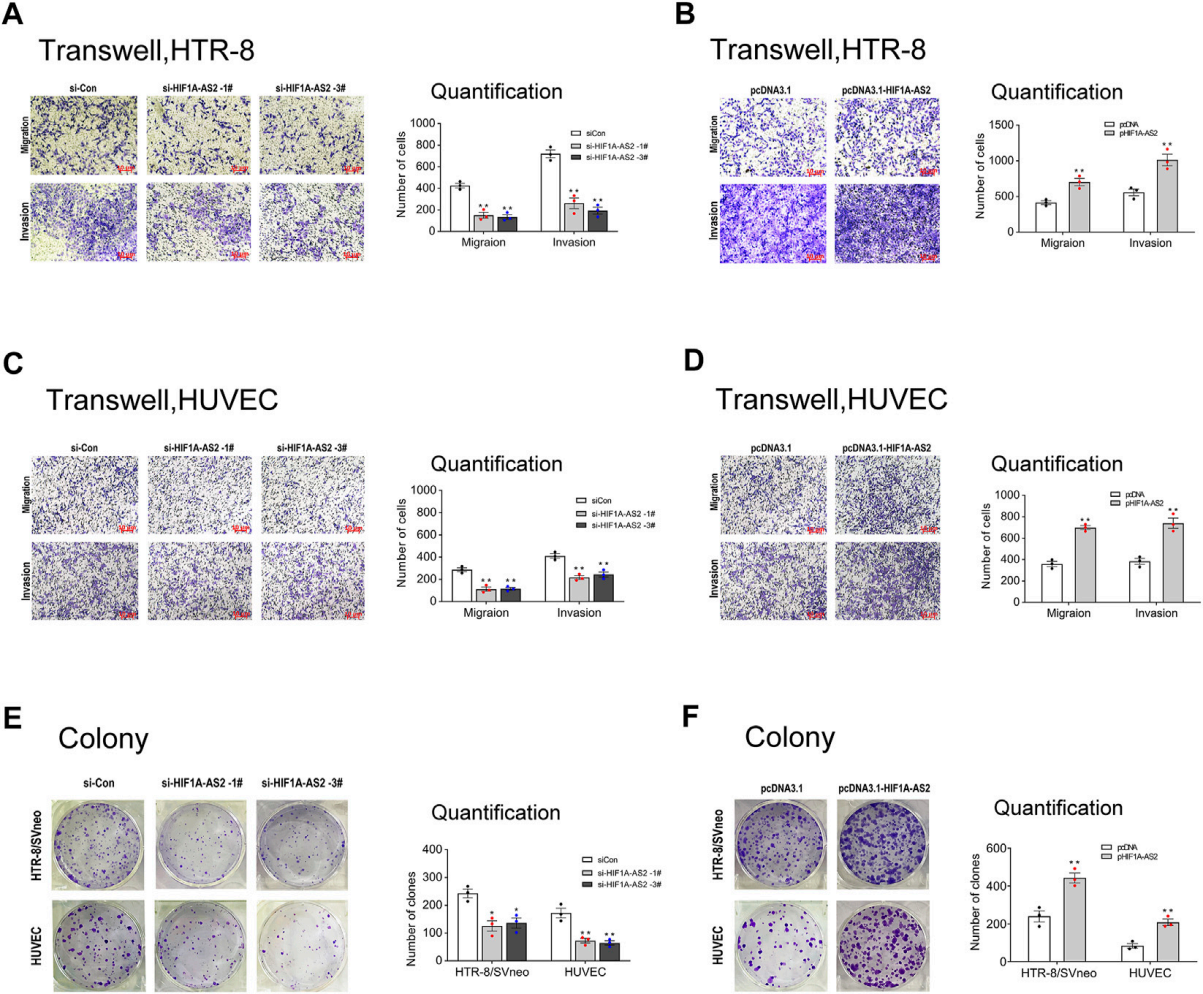
*HIF1A-AS2* promotes trophoblast proliferation, migration and invasion *in vitro*. Transwell assays showed the migratory and invasive abilities of si*HIF1A-AS2*-treated in HTR-8/SVneo **(A)** and HUVECs **(C).** The quantity of cells was markedly decreased compared to siCon control. With the treatment of overexpressed *HIF1A-AS2,* the 2 cell lines showed significant increase than that in pcDNA control **(B,D)**. Colony formation assay to assess the ability of proliferation in HTR-8/SVneo and HUVEC cells, transfected with siRNAs or plasmid. Silenced *HIF1A-AS2* expression in HTR-8/SVneo and HUVEC cells **(E)** and overexpressed-*HIF1A-AS2* **(F)**. The statistics of the relevant data is on the right side of the corresponding graph. Error bars are mean with SEM of technical replicates (*n* = 3), *p < 0.05, **p < 0.01; significance by Student’s t test.

In response to abnormal *HIF1A-AS2* expression levels, the cellular activities of trophoblasts were expected to exhibit corresponding changes. To verify the association, transwell experiments were conducted to evaluate the invasion and migration abilities of HTR-8/SVneo and HUVEC cells. For establishing blood flow between maternal and fetal systems, appropriate cellular motility and adhesion of trophoblasts is essential, whereas the impaired placental vascular development of PE patient is induced by suppressed trophoblast activities. The silencing of *HIF1A-AS2* could inhibit the invasion and migration of HTR-8/SVneo and HUVECs (Figures 2A,C), while the upregulated *HIF1A-AS2* expression presented causal relationship with boosted trophoblast invasion and migration (Figures 2B,D).

Next, in the colony formation assay, when *HIF1A-AS2* was silenced, both tested cell lines showed decreased clone numbers (Figure 2E). Conversely, the elevated *HIF1A-AS2* expression facilitated cell number increases *in vitro* (Figure 2F), confirming the positive relationship between *HIF1A-AS2* expression and the proliferation of the tested cell lines. Thus, verified by these noticeable findings, *HIF1A-AS2* could effectively modulate the proliferation, invasion and migration of trophoblast cells and umbilical vein endothelial cells.

### *HIF1A-AS2* promotes angiogenesis *in vitro* and *in vivo*

**FIGURE 3:**
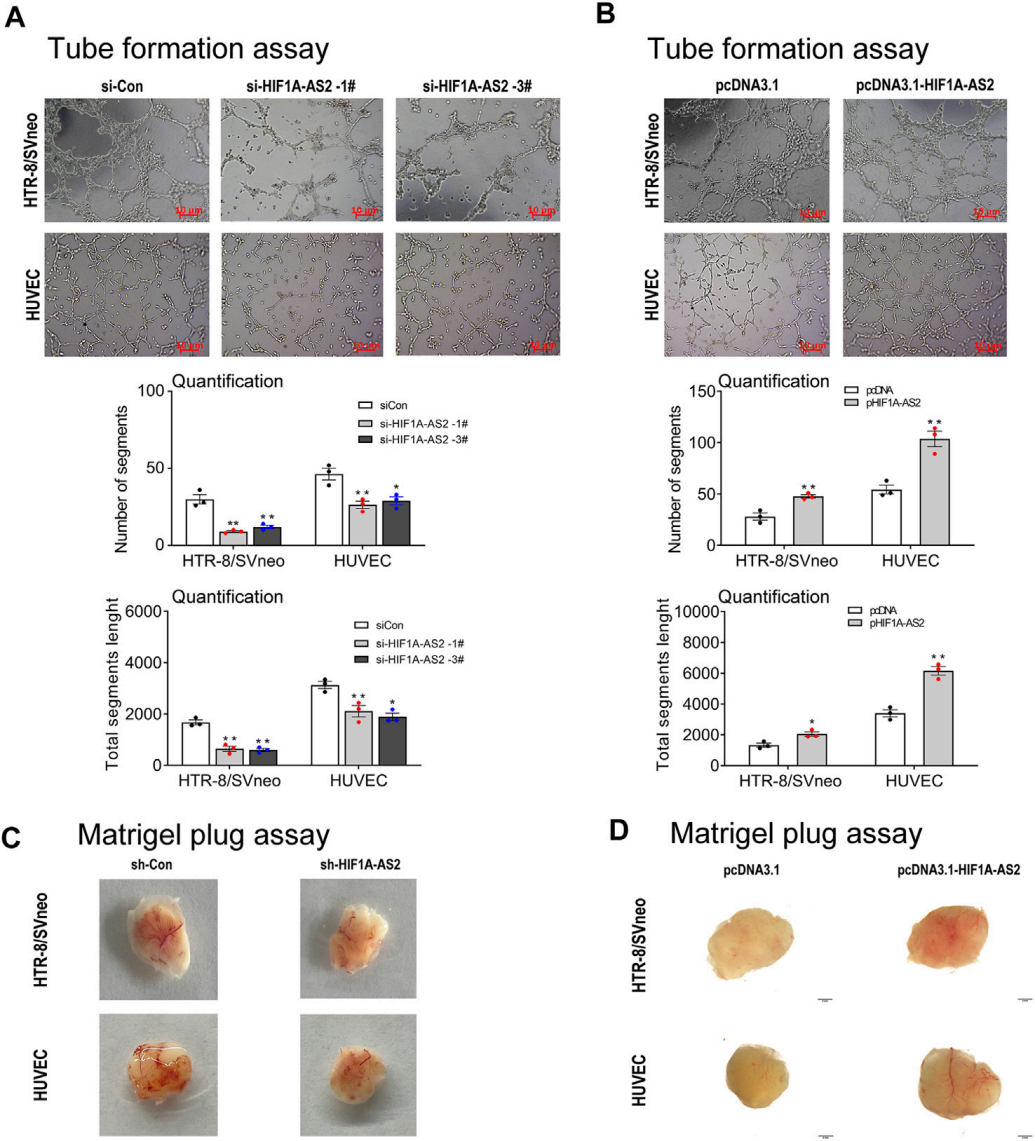
*HIF1A-AS2* affects angiogenesis of HTR-8/SVneo trophoblast and HUVEC cells *in vitro* and *in vivo*. Consequent to network formation, the number of segments and total segments length of the network were decreased in *HIF1A-AS2* with siRNAs **(A)** and increased in overexpressed with pcDNA3.1-*HIF1A-AS2* **(B)** in HTR-8/SVneo and HUVECs, respectively. The quantity of cells is shown below the corresponding graph. The Matrigel plug assay showed that *HIF1A-AS2*-related siRNA inhibited angiogenesis in nude mice **(C)**, and overexpression of *HIF1A-AS2* promoted angiogenesis **(D)**. Error bars are mean with SEM of technical replicates (*n* = 3). *p < 0.05, **p < 0.01; significance by Student’s t test.

Abnormal development of uterine spiral artery remodeling in placenta is one of the main predisposing conditions of PE, which can induce ischemia, hypoxia, oxidative stress, and excessive release of various cytokines into the maternal blood circulation, leading to systemic Inflammatory immune overactivation and vascular endothelial damage. In order to recognize the association between *HIF1A-AS2* and vascular remodeling, for the two essential cells in the placental blood vessels recasting, tube formation assay was applied. *HIF1A-AS2* silencing inhibited the tube formation ability of HTR-8/SVneo cells and HUVECs (Figure 3A). Consistently, the numbers of segments and total segments lengths were both decreased compared to the controls. *HIF1A-AS2* overexpression resulted in the opposite trend (Figure 3B). The presence of red plugs in the tested cell lines was significantly different from that of the vector control cell sample, confirming that enhanced *HIF1A-AS2* expression promoted the angiogenic activities of HTR-8/SVneo and HUVECs cells (Figure 3D). Knock-down of *HIF1A-AS2* led to the opposite outcome (Figure 3C). To summarize these observations, *HIF1A-AS2* played a pivotal role in proangiogenic activities of HTR-8/SVneo cells and HUVECs.

### *HIF1A-AS2* can be activated by the transcription factor forkhead box P1

**FIGURE 4:**
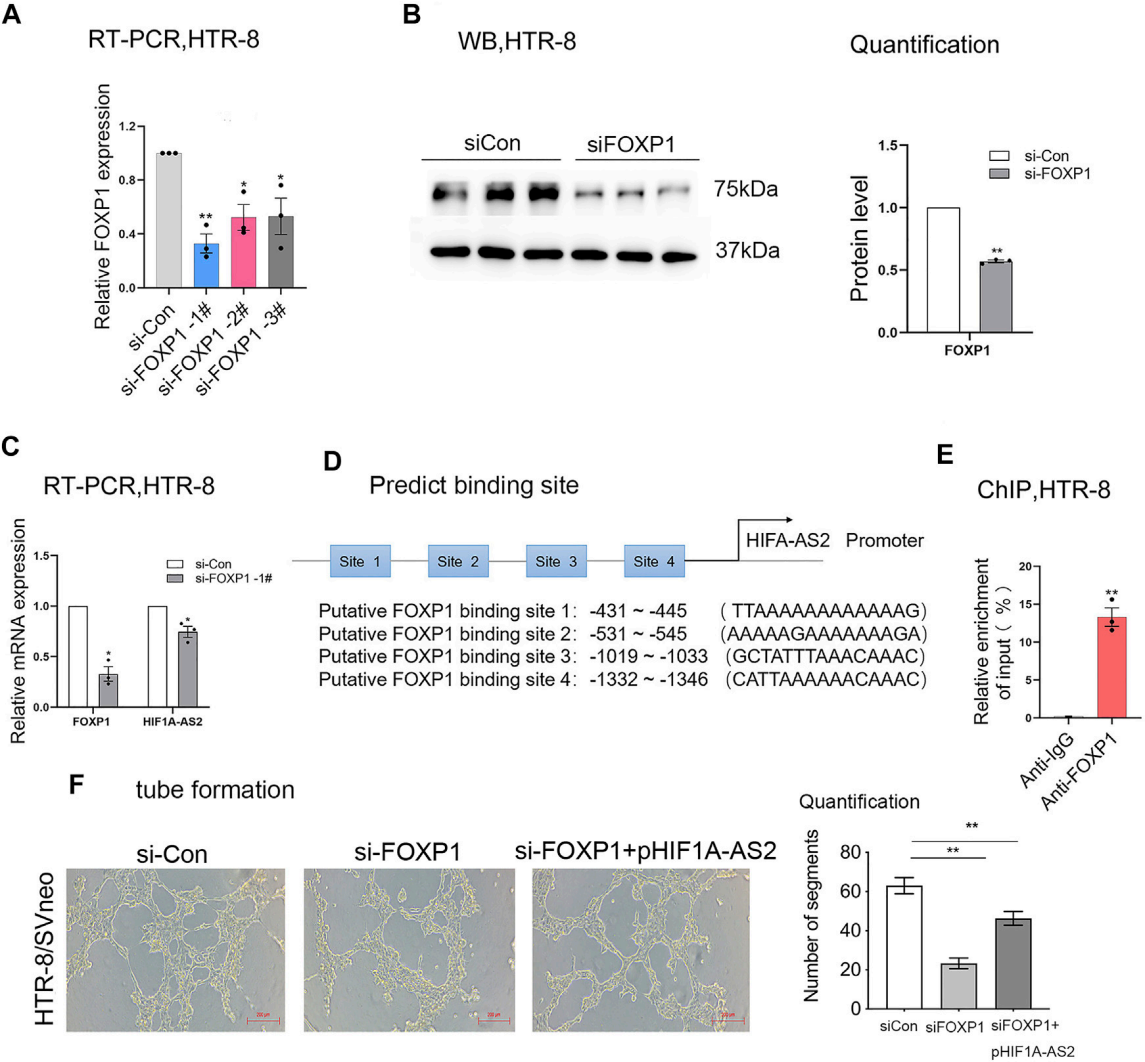
*HIF1A-AS2* interacts with the transcription factor FOXP1. FOXP1 expression level was detected at RNA and protein level by qPCR **(A,C)** and Western blotting **(B)**, respectively, after FOXP1-specific siRNAs were transfected into HTR-8/SVneo. After the treatment with siRNAs against *HIF1A-AS2,* the lncRNA expression was tested by qPCR **(C)**. The putative binding sites of FOXP1 on *HIF1A-AS2* promoter region, and four potentially relevant binding sequences were acquired by using the JASPAR website **(D)**. ChIP assay confirmed the binding of FOXP1to the promoter of *HIF1A-AS2* in HTR-8/SVneo. qPCR data were compared with IgG negative control **(E)**. Error bars are mean with SEM of technical replicates (*n* = 3). *p < 0.05, **p < 0.01; significance by Student’s t test. Performing network formation, cells transfected with FOXP1 siRNAs and HIF1A-AS2 plasmid showed an increase in node numbers as compared with silencing of FOXP1 in HTR-8/SVneo **(F)**. Error bars are mean with SEM of technical replicates (*n* = 3). *p < 0.05 and **p < 0.01; significance by one-way ANOVA with Tukey post-test. All data are representative of at least two independent experiments.

The expression patterns of various lncRNAs had been proved to be mediated by transcription factors in previous research (Sato et al., 2010; Xu et al., 2020). Relying on the bioinformatic approach, the JASPAR database (http://jaspar2014.genereg.net) could be utilized to identify the candidate factors activating or inactivating *HIF1A-AS2* expression. As listed in Table 1, in *HIF1A-AS2* promoter region, there were 15 Forkhead box P1 (FOXP1)-binding putative sites, implying that *HIF1A-AS2* may be regulated by transcription factor FOXP1 in trophoblast cells. In our previous study, we analyzed the expression levels of FOXP1 in 64 pairs of PE samples and adjacent normal samples with normalization to GAPDH by quantitative real-time PCR assay. The expression of FOXP1 was significantly downregulated in PE placenta compared with that in control. The same trend was also obtained in the animal model of preeclampsia (Xu et al., 2022). Subsequently, FOXP1 expression at both RNA and protein levels in the HTR-8/SVneo were silenced with siRNAs (Figures 4A,B), and in response to the manipulation, *HIF1A-AS2* expression showed obvious decreases (Figure 4C). The putative binding sites of FOXP1 on HIF1A-AS2 promoter region, and four potentially relevant binding sequences were acquired by using the JASPAR website (Figure 4D).

**TABLE 1:** Putative FOXP1-binding sites in the *HIF1A-AS2* promoter region by JASPAR.

Model ID	Model name	Score	Relative score	Start	End	Strand	Predicted site sequence
MA0481.1	FOXP1	7.935	0.801261316011212	428	442	1	AACTTAAAAAAAAAA
MA0481.1	FOXP1	8.473	0.810077265208495	429	443	1	ACTTAAAAAAAAAAA
MA0481.1	FOXP1	10.967	0.850945252751287	430	444	1	CTTAAAAAAAAAAAA
MA0481.1	FOXP1	11.268	0.855877596075418	431	445	1	TTAAAAAAAAAAAAG
MA0481.1	FOXP1	8.241	0.8062755919487	433	447	1	AAAAAAAAAAAAGTA
MA0481.1	FOXP1	14.607	0.910592195275652	455	469	−1	AAAATGTAAAGAAAA
MA0481.1	FOXP1	8.596	0.812092807497093	526	540	1	TTGAAAAAAAGAAAA
MA0481.1	FOXP1	9.004	0.818778508747076	530	544	1	AAAAAAGAAAAAAAG
MA0481.1	FOXP1	11.034	0.85204314977028	531	545	1	AAAAAGAAAAAAAGA
MA0481.1	FOXP1	10.921	0.850191472708397	532	546	1	AAAAGAAAAAAAGAG
MA0481.1	FOXP1	11.089	0.852944408517214	1011	1025	−1	AAATAGCAAACAGAA
MA0481.1	FOXP1	8.881	0.816762966458478	1019	1033	1	GCTATTTAAACAAAC
MA0481.1	FOXP1	8.869	0.81656632818642	1030	1044	1	AAACAGTTAACAATG
MA0481.1	FOXP1	12.273	0.872346051360304	1332	1346	−1	CATTAAAAAACAAAC
MA0481.1	FOXP1	8.944	0.817795317386785	1797	1811	−1	GGTCACAAAACAAAA

Next, the interpretation of ChIP assays supported that the binding of FOXP1 to the *HIF1A-AS2* promoter region could upregulate the lncRNA at RNA level (Figure 4E). Then we performed tube formation assay in HTR-8/SVneo, the results showed that the inhibition of FOXP1 and upregulation of *HIF1A-AS2* can promote antiogenesis compare with silencing of FOXP1 (Figure 4F). Therefore, FOXP1 was suggested to be the most potent candidate factor for the upstream regulation on *HIF1A-AS2* activation.

### *HIF1A-AS2* could regulate angiopoietin like 4 by binding to lamin A/C

**FIGURE 5:**
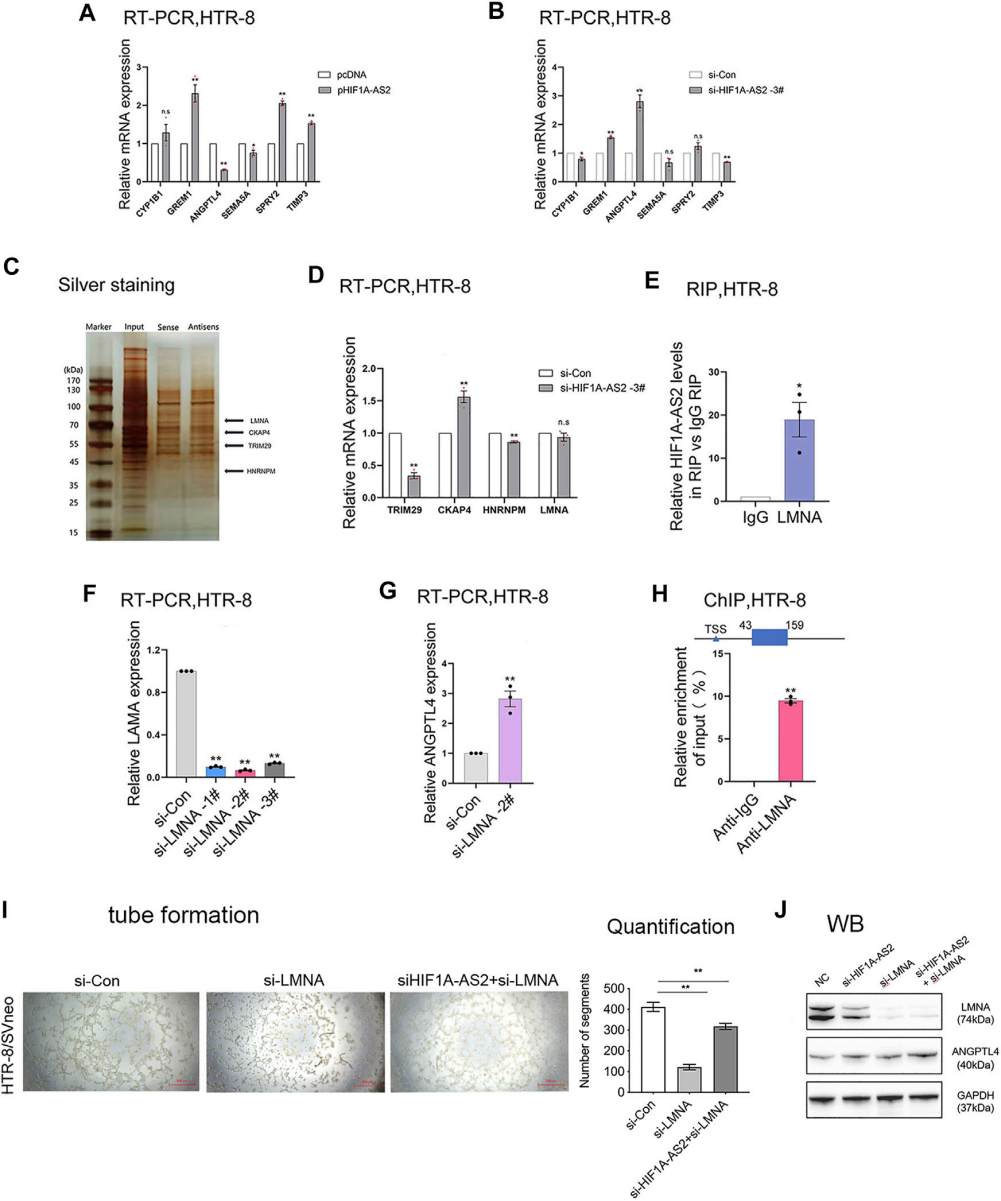
*HIF1A-AS2* binds to LMNA to inhibit ANGPTL4 expression. qPCR analysis of HTR-8/SVneo cells uncovered the relative mRNA expression of vascularized genes, with **(A)** *HIF1A-AS2* overexpression or **(B)** knockdown by si*HIF1A-AS2*. RNA pull down, silver staining and mass spectrometry analysis were combined to analyze several potential binding proteins **(C)**. After transfected with si*HIF1A-AS*2, the changes in the mRNA expression of several possible binding proteins were analyzed by qPCR **(D)**. RIP results showed that *HIF1A-AS*2 could bind LMNA in HTR-8/SVneo cells **(E)**. After LMNA knockdown, the expression of ANGPTL4 was increased at RNA levels **(F,G)**. Error bars are mean with SEM of technical replicates (*n* = 3). *p < 0.05, **p < 0.01; significance by Student’s t test. ChIP assays revealed the enrichment of LMNA in ANGPTL4 promoter region **(H)**. Consequent to tube formation, the number of segments and total segments length of the tube were decreased in the inhibition of *HIF1A-AS2* and LMNA **(I)**; ANGPTL4 and LMNA expression levels were detected at protein level by WB in HTR-8/SVneo **(J)**. Error bars are mean with SEM of technical replicates (*n* = 3). *p < 0.05 and **p < 0.01; significance by one-way ANOVA with Tukey post-test. All data are representative of at least two independent experiments.

Our research group had carried out RNA transcriptome sequencing for the siRNA (siCon) and si*HIF1A-AS2*-transfected HTR-8/SVneo cells with the presence of negative control, in order to identify the downstream influences of altered *HIF1A-AS2* expression (Wu et al., 2019). Relying on the Gene Ontology (GO) and Kyoto Encyclopedia of Genes and Genomes (KEGG) databases, the biological pathways relevant to *HIF1A-AS2* activation could be identified. In response to *HIF1A-AS2* silencing, a bunch of genes responsible for vascular development were significantly affected in their expression levels (Wu et al., 2019). Through qPCR, Angiopoietin Like 4 (ANGPTL4), a transcript related to vascular development was selected, the expression of which showed significant downregulation based upon *HIF1A-AS2* overexpression (Figure 5A) and upregulation based upon *HIF1A-AS2* knockdown in HTR-8/SVneo (Figure 5B).

Our previous studies had clarified the distribution of *HIF1A-AS2* RNA, mainly in the nucleus of HTR-8/SVneo cells (Wu et al., 2019). Accordingly, *HIF1A-AS2* might have functions in transcriptional regulation *via* recruiting RNA-binding proteins. To examine the prediction, RNA pull down assay and mass spectrometry analysis were conducted, and the results showed that *HIF1A-AS2* might interact with Lamin A/C (LMNA) (Figure 5C). However, qPCR assays found that there was almost no change in LMNA expression at the RNA level (Figure 5D) after knocked down *HIF1A-AS2*. Subsequently, relying on the results of RNA immunoprecipitation (RIP) analysis, it was also proved that *HIF1A-AS2* could recruit LMNA in HTR-8/SVneo (Figure 5E).

The protein products of the LMNA gene, Lamin A/C proteins, are essential for the normal functions of the nuclear lamina due to their multiple cellular functions, including the modulation of DNA transcription, the maintenance of chromatin organization and nuclear stability, the regulation of cell cycle, and the initiation of apoptosis (Nishikawa et al., 2021). Then, our results revealed that the expression of ANGPTL4 was increased after down-regulating LMNA with specific-siRNAs at RNA level (Figures 5F,G). Moreover, we performed ChIP assays and revealed that LMNA could directly interact with ANGPTL4 promoter regions (Figure 5H). If considering the downstream interactions as an integrated mechanism, it suggested that *HIF1A-AS2* might regulate the expression of ANGPTL4 in trophoblasts through interacting with LMNA. Then we performed tube formation assay, and the results indicated that the inhibition of *HIF1A-AS2* and LNMA can promote antiogenesis compare with silencing of LNMA (Figure 5I). Also, the western blotting assays were conducted, and the expression of ANGPTL4 was significantly elevated in the inhibition of HIF1A-AS2 and LMNA compare with that in the silencing of LNMA (Figure 5J).

### Angiopoietin like 4 expression is significantly elevated in preeclampsia placental tissues

**FIGURE 6:**
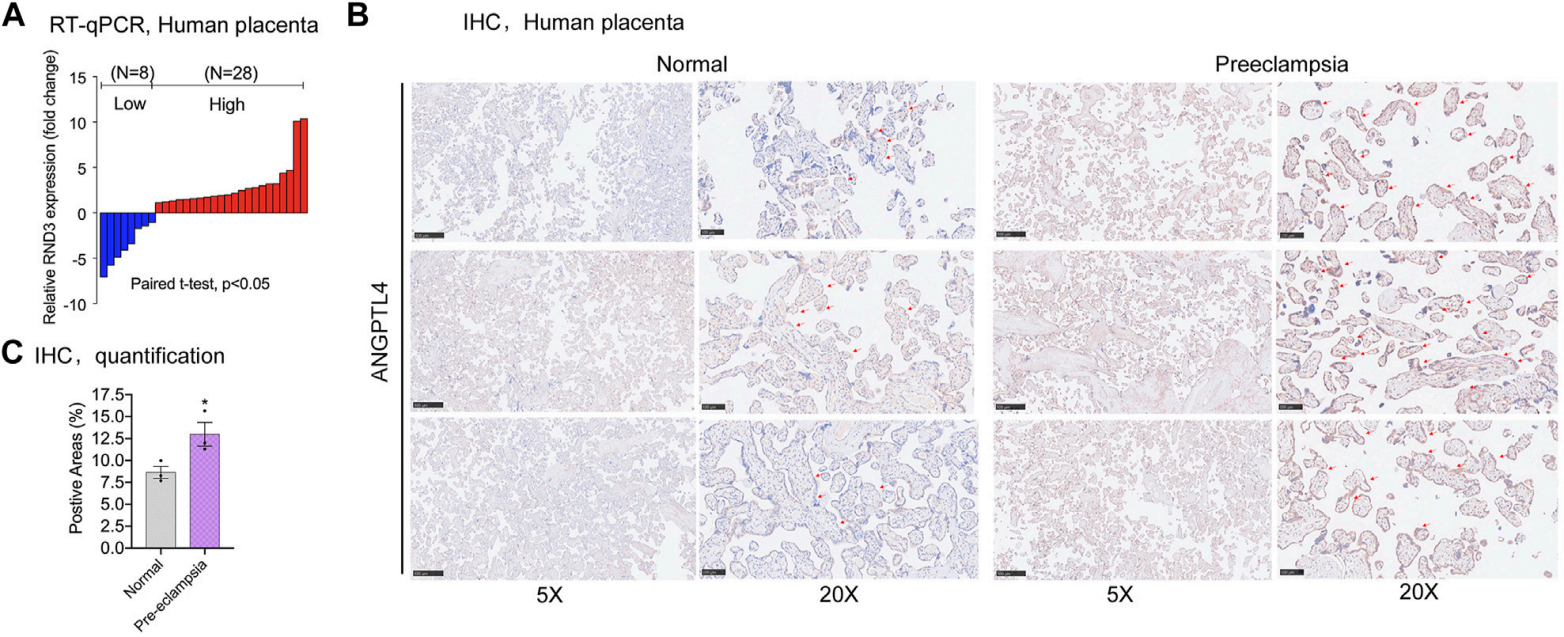
Expression of ANGPTL4 in preeclampsia placental tissues. qPCR results showed the fold change of placental tissue in PE patients compared to healthy pregnant women (*n* = 34) **(A)**. The abundance of ANGPTL4 protein was detected by immunohistochemistry **(B)**, quantification was performed by positive areas **(C)**. All data are representative of at least three independent experiments. *p < 0.05, **p < 0.01; significance by Student’s t test.

To evaluate the relevance between ANGPTL4 and PE, mRNA expression levels of ANGPTL4 were compared between placental tissue samples from severe PE patients and healthy pregnant participants. qPCR assays were carried out, and the results suggested increased ANGPTL4 mRNA expression in severe PE tissue samples compared with the control (Figure 6A). Furthermore, we performed immunohistochemistry assay and consequently confirmed that ANGPTL4 expression levels of the PE-positive placental samples were elevated at protein level (Figures 6B,C). In conclusion, for the patients of severe PE, ANGPTL4 expression level had significant negative correlation with that of *HIF1A-AS2* in their placental tissue samples.

## Discussion

Among the large variety of non-coding RNAs existing in human genome, the two major types, microRNAs (miRNAs) and lncRNAs (>200 nucleotides), are currently sorted by the lengths, and their total number is approximately 16.000. Due to the necessity of their functions to maintain the normal running of relevant biological processes, the correlations between the abnormal expression of specific lncRNAs and the occurrences of diseases had been increasingly noticed (Sun et al., 2018; Statello et al., 2021). To be specific, the interest of our group was the association between the candidate lncRNAs and the pathogenesis of PE. Our published papers had identified some PE-related lncRNAs, including *TUG1, PVT1*, and *HOXA11-AS1* (Xu et al., 2017; Xu et al., 2018a; Xu et al., 2018b). Based on the precedent studies, this study aimed to verify the relationship between *HIF1A-AS2* and PE, thereby revealing more details of the pathogenetic mechanisms at molecular level and proposing the new candidates for further analyses to evaluate their potential in clinical practice against PE.

Multiple preceding studies had revealed that *HIF1A-AS2* could effectively participate in the pathogenetic mechanisms of various malignancies (Wang et al., 2019a; Wang et al., 2019b; Si et al., 2021). Combined with our existing research work, the PE-related functions of *HIF1A-AS2* could be preliminarily identified. To obtain more details at molecular level, this study was designed to further evaluate the influences of abnormal *HIF1A-AS2* expression on HTR-8/SVneo and HUVEC. *In vitro*, the silencing of *HIF1A-AS2* expression resulted in remarkably suppressed proliferation, invasion, migration and vascularization of trophoblast cells, and consistently, overexpressed *HIF1A-AS2* also induced significant changes in these cellular activities at the opposite direction. *In vivo*, we observed that upregulation of *HIF1A-AS2* could promote angiogenesis in trophoblast cells and vascular endothelial cells. The observations supported the effective participance of *HIF1A-AS2* in the development of PE, which depended on the regulatory functions of the lncRNA, and might indicate its clinical significance in PE. Overall, our further analyses on the underlying molecular mechanisms should be promising.

Next, we performed high-throughput sequencing (RNA-seq) to explore the downstream objects of *HIF1A-AS2*. According to the *in-vitro* tests, after suppression of *HIF1A-AS2*, noticeable increases of the cellular inactivator ANGPTL4 could be detected at both the mRNA and proteins level. ANGPTL4, located on chromosome 19p13.2, encodes a peptide hormone belonging to the family of adipokines. Its protein product has a variety of biological functions, including the modulatory effects on cell cycle progression, metastasis and angiogenesis (Guo et al., 2014; Yang et al., 2020). However, the exact mechanisms establishing the association between ANGPTL4, and preeclampsia remains a mystery. For the accessibility between ANGPTL4 and *HIF1A-AS2,* our previous study had reported the predominant nuclear localization of *HIF1A-AS2* in trophoblast cells (Wu et al., 2019). The subsequent mechanism experiments included RIP assay, RNA pull down and mass spectrometry analysis. The results provided more details to confirm the interaction between *HIF1A-AS2* and LMNA in trophoblasts. According to the findings, in the pathogenetic mechanism of PE, a part of the downstream pathways for *HIF1A-AS2* might rely on its binding to LMNA, thereby suppressing ANGPTL4 expression in trophoblasts.

Mounting studies stated that transcript factor could activate/inactivate lncRNA transcription further regulating target expression which accelerates the progression of PE (Ma et al., 2014; Ghosh et al., 2018; Huh et al., 2019). For example, Xu et al. (2019) reported that KLF5 could act as transcriptional factor and modulate lncRNA 00346-prompted gastric cancer progression. Reported by our published work, the transcript factor FOXP1 could contribute to the activation of *AGAP2-AS1* expression in trophoblasts (Xu et al., 2020). Accordingly, for this study, the bioinformatic analysis also suggested that FOXP1 might promote *HIF1A-AS2* expression at transcriptional level (Xu et al., 2020). To verify this conjecture, ChIP-PCR results demonstrated that FOXP1 could directly bind to the promoter region of *HIF1A-AS2* in HTR-8/SVneo. Furthermore, FOXP1 upregulation could enhance *HIF1A-AS2* transcription in trophoblasts. Together, these findings established that *HIF1A-AS2* could be activated by FOXP1, emphasizing the complicity of the upstream regulation on the expression patterns of lncRNAs.

## Conclusion

**FIGURE 7:**
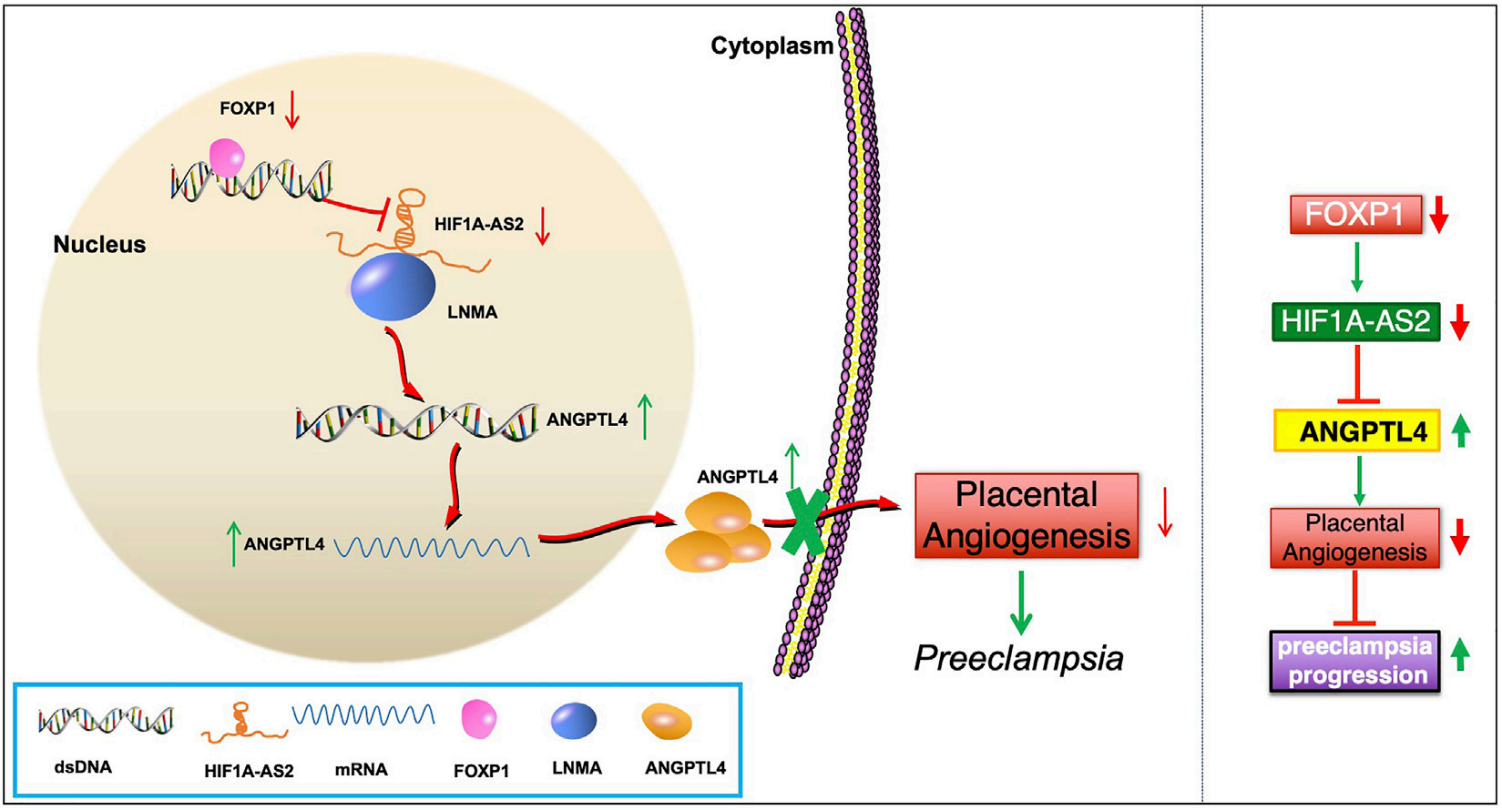
Schematic of the regulatory pathway.

To summarize the discoveries in this study, among the pathogenetic mechanisms of PE, the abnormal *HIF1A-AS2* downregulation can induce the enhancement of ANGPTL4 expression *via* the LMNA-mediated regulatory pathway, resulting in aggravated dysfunction of angiogenesis (Figure 7). Thus, *HIF1A-AS2* has the potential to be investigated as a candidate molecular target which may facilitate the improvement of clinical strategies against PE. However, for the limitation of this study, the potential biological phenotypes of *HIF1A-AS2* have not been completely explored, while the identification of other achievable downstream and regulatory mechanisms could still be expected. Further studies are required to illuminate other relevant upstream and downstream pathways, through which *HIF1A-AS2* can exert its regulatory functions on the cellular activities of trophoblasts in PE.

## Materials and methods

### Cell culture

Human trophoblast cell (HTR-8/SVneo) and human umbilical vein endothelial cells HUVECs were offered by the Type Culture Collection of the Chinese Academy of Sciences (Shanghai, China). RPMI 1640 medium, added by 10% fetal bovine serum (FBS, GIBCO, United States) and antibiotics (100 U/ml of penicillin and 100 mg/ml of streptomycin), was the choice to culture HTR-8/SVneo cell. HUVEC cells were cultured in ECM medium. The temperature set for cell growth was 37.0°C, with high humidity and 5% CO_**2**_ in the incubating condition.

### Cell transfection

DNA Midiprep kits (QIAGEN, Hilden, Germany) were chosen, which enable the plasmid vectors (pcDNA3.1-*HIF1A-AS2*) to arrayed. The sequences of siRNAs, including *HIF1A-AS2*-specific siRNAs, FOXP1 siRNA, LAMA siRNA and scrambled siCon, were designed by Gene Pharma; their details were listed in Supplementary Table S1. After HUVECs or HTR-8/SVneo cells were inoculated on 12-well plates, transfection could be carried out when the degree of cell fusion reached 70%–80%. Reagent Lipofectamine 2000 (Invitrogen, United States) was applied for siRNA transfection. FuGENE HD Transfection reagent (Roche, Pennsburg, Germany) was applied to treat the plasmid. The processing steps complied with manufacturer’s instructions. After 24 h, the treated cells were collected for further analyses.

### Extract RNA and qPCR

FastPure Cell/Tissue Total RNA Isolation Kit V2 (Vazyme, Nanjing, China) was chosen to conduct total RNA extraction. The following measurements of RNA concentration and purity was achieved with Onedrop. RNA was reversely transcribed into cDNA using HiScript®III RT SuperMix for qPCR (+gDNA Wiper (Vazyme, Nanjing, China). ChamQ SYBR qPCR Master Mix (High ROX Premixed) (Vazyme, Nanjing, China) was applied to detect the expression of *HIF1A-AS2* and LAMA, and the mixture was configured according to the instructions of StepOnePlus™ (Thermo Fisher Scientific, Waltham, MA, United States) instrument. The qPCR data were analyzed by StepOne™ software v2.3. The PCR primers were listed in Supplementary Table S1.

### Colony formation assays

For colony formation capability, each cell sample had been treated with a specific siRNA or the plasmid before the seeding. Each well of the 6-well plate was filled with 600, 800 or 1,000 cells and had the addition of 2 ml 10% FBS complete medium. About 10–14 days after the formation of colony, with the fixation used methanol and the staining used 0.1% crystal violet. The capability of colony formation was quantified as the number of stained colonies.

### Migration and invasion assays

To assess cell migration and invasion, the transwell chamber (pore size 8 μm) was placed in a 24 well plate. Briefly, the 4–6 × 10^**4**^ cells suspended in 300 μl empty medium were seeded into the chamber inside, and 700 μl complete medium containing 10% FBS was infused into the chamber outside. The diluted Matrigel Matrix (Corning, United States) was coated or uncoated on top of the membrane for invasion or migration assays, respectively. Culturing for 24–48 h, the cells below the membrane were fixed with methanol and stained with 0.5% crystal violet. Under the light microscopy, from each chamber, 3-5 areas were picked up by random selection for photographing and data recording.

### Tube formation assays

The precooled Matrigel Matrix (Corning, United States) was added to the wells in the 96-well plates, in order to achieve polymerization by incubation at 37°C for half an hour. HUVECs or HTR-8/SVneo cells were inoculated onto 12-well plates, followed by being transfected with 3 nM siRNAs or 2 μg plasmid for 24 h. The treated HUVECs or HTR-8/SVneo were digested. Subsequently, each sample was suspended in 150 μl conditioned medium and then transferred to a coated 96-well plate (4 × 10^**4**^ cells/well or 6 × 10^**4**^ cells/well). The incubation conditions were set at 37°C in 5% CO_**2**_ for 8–16 h. Under light microscopy, photographs were taken. The analysis of the formed tube was conducted by an ImageJ Angiogenesis Analyzer.

### Western blotting assays

Total proteins in cells or tissues were extracted with cold lysates and separated by 10% TGX FastCast acrylamide gel (Bio-Rad, United States), followed by being transferred to 0.45 μm PVDF membranes (Millipore, United States). Subsequently, the PVDF membrane was sealed with a blocking solution for 15 min at room temperature. The appropriate dilution antibody (FOXP1, CST, Catalog No. D35D10, United States) and anti-GAPDH antibody (Proteintech, Catalog No. 10494-1-AP, United States) were applied for overnight incubation on a shaker in a 4°C fridge. After washing, the secondary antibody was applied for 1 h incubation. The chemiluminescence (Thermo) fluid was applied for exposure. Quantity One software (Bio-RAD) read the protein bands and interpreted the patterns into numeric data.

### RNA pull down and mass spectrometry

After the linearization of the plasmid templates, the mMESSAGE mMACHINE T7 Transcription Kit (Life Technologies, United States) was applied for *in vitro* transcription. Subsequently, complying with the manufacturer’s instructions, the major steps of RNA pull down assay was conducted as below. A single biotinylated nucleotide was attached to label RNA 3′terminus with T4 RNA ligase. Labeled *HIF1A-AS2* capture was achieved with streptavidin magnetic beads, which was then incubated with cell protein lysates from HTR-8/SVneo. After silver staining, the protein eluted solution was ready for mass spectrometry analysis on Q Exactive mass spectrometer (Proxeon Biosystems, now Thermo Fisher Scientific), Analysis of the mass spectrometry data are listed in Supplementary Table S2.

### Chromatin immunoprecipitation assays

EZ-Magna ChIP A kit (Catalog No. 17–408, Merck & Millipore) was applied to for the ChIP assay. The steps complied with the manufacturer’s instructions. After growth, HTR-8/SVneo cells were treated with formaldehyde, which crosslinks proteins to DNA to ensure co-precipitation. Then, the cells were broken open, which allowed the sonication to shear the chromatins into 200-1,000 bp DNA pieces. Immunoprecipitation was performed using primary antibodies or control IgG. Next, after the chromatin proteins were removed by DNA purification, qPCR was applied. The detailed information about specific ChIP primers were exhibited as Supplementary Table S1 **.**

### RNA immunoprecipitation assays

Following the procedures introduced by Xu et al. (2018a), RIP assays were set up. The producer of the LMNA antibodies was Millipore (Billerica, MA, Catalog No. 17–701 Merck & Millipore United States). For statistical analyses, qPCR was conducted to interpret the presence of *HIF1A-AS2* and IgG into numeric data.

### Matrigel plug assay in nude mice

All experiments on mice complied with the guidance of Laboratory Animal Management Regulations, the national law of China and the instruction of the local authority. The approval of Zoopery was issued by the Ethics Committee of Nanjing Medical University Animal Welfare (IACUC-2106050). The plug assay was carried out as the conventional method reported by previous studies (Aktas et al., 2017; Ding et al., 2019). Briefly, female BALB/c nude mice (4–6 weeks after birth) were qualified for the experiments. The cells were resuspended with 0.2 ml serum-free medium and evenly mixed with equal volume of High Concentration Matrigel (BD Biosciences). Immediately, the mixture was injected subcutaneously. After the injection, 2 weeks later, Matrigel plugs were removed from the euthanized mice.

### Statistical analysis

The data came from three independent experiments. GraphPad Prism version 8.0 (GraphPad Software) conducted the statistical calculations. Adobe Photoshop 2020 contributed to the graphic presentation. The evaluation of results passed two-tailed Student’s t test and expressed as mean ± S.E.M. **p < 0.01, *p < 0.05 were considered significant.

## Data Availability

The original contributions presented in the study are included in the article/Supplementary Materials, further inquiries can be directed to the corresponding authors.

## Supplementary material

Supplementary Table S1 The list of PCR/ChIP-PCR primers and siRNAs sequences.

Supplementary Table S2 Analysis of the mass spectrometry data.

## Abbreviations

lncRNA, long non-coding RNA; PE, Preeclampsia; ANGPTL4, Angiopoietin-like 4; LMNA, Lamin A/C; RIP, RNA immunoprecipitation; ChIP, Chromatin immunoprecipitation.
