# Supplementary material for: A novel regulated network mediated by downregulation HIF1A-AS2 lncRNA impairs placental angiogenesis by promoting ANGPTL4 expression in preeclampsia
Source: Front Cell Dev Biol. 2022 Aug 9;10:837000. doi: 10.3389/fcell.2022.837000 (PMC9396278; doi:10.3389/fcell.2022.837000)
Supplement: Supplementary file 2 [file Table1.docx]

| **Supplementary Table 1** |  |  | | |
| --- | --- | --- | --- | --- |
| **Real-time PCR primer sequences** | | |  |  |
| **Gene** | **Forward Primer** | **Reverse Primer** | | |
| HIF1A-AS2 | 5’-TCTGTGGCTCAGTTCCTTTTGT-3' | 5’-ATGTAGGAAGTGCCAGAGCC-3' | | |
| GAPDH | 5’-AGCCACATCGCTCAGACAC-3' | 5’-GCCCAATACGACCAAATCC-3' | | |
| FOXP1 | 5’-GTTGCAGTCCTGTGGCATTA-3' | 5’-AGACCGCCGCACTCTAGTAA-3' | | |
| LMNA | 5’-TGGGCTGTCTCTGTCTGTTG-3' | 5’-GCTCTGTCATCACTCAGGCA -3' | | |
| TRIM29 | 5’-cgccacgttgagaagatgt-3' | 5’-gacgagggctggtatgatgt -3' | | |
| CKAP4 | 5’-cagcaccgtggaatcactc-3' | 5’-ttgaccgagtatgcaaccaa -3' | | |
| HNRNPM | 5’-agctgcggaagtcctaaaca-3' | 5’-ttccaagtcttccagcctgt -3' | | |
| ANGPTL4 | 5’-CACACGACTGTGATCCGATT-3' | 5’-AGCACCGCTCATCCTCTTAG -3' | | |
| TIMP3 | 5’-CACACGACTGTGATCCGATT-3' | 5’-AGAGCATGTCGGTCCAGAGA -3' | | |
| SPRY2 | 5’-GCCTACTGTCGTCCCAAGAC-3' | 5’-AGCTGACCGTGCTTATGGAT -3' | | |
| SEMA5A | 5’-CCTGGTTACGGGAGTTCAGA-3' | 5’-TCCTTTGATTTGCCTTTGCT -3' | | |
| CYP1B1 | 5’-CAGAATTGGATCAGGTCGTG-3' | 5’-ACAGAGGTGTTGGCAGTGGT -3' | | |
| GREM1 | 5’-AACTTGGCCTACTGGCAATG-3' | 5’-CCTTGATCGGCAACTGAATC -3' | | |
|  |  |  | | |
|  |  |  | | |
| **CHIP-PCR primer sequences** | | |  |  |
| HIF1A-AS2-P1 | 5’- gcctggtccacagaagatgt -3' | 5’- tgttctgcctaccctgttgg -3' | | |
| ANGPTL4 | 5’- cacacgactgtgatccgatt -3' | 5’- agcaccgctcatcctcttag -3' | | |
|  |  |  | | |
| **FXOP1 siRNA sequence (human)** | | |  |  |
| **1# sense 5’- CTCAGTCCACACTCCCAAA -3’** | | |  |  |
| **2# sense 5’- CCACAGAGCTTACCTCATA -3’** | | |  |  |
| **3# sense 5’- CTGGTTCACACGAATGTTT -3’** | | |  |  |
|  | | |  |  |
| **HIF1A-AS2 siRNA sequence (human)** | | |  |  |
| **1# sense 5'- GGCUCUGGCACUUCCUACAUAAUUU -3’** | | |  |  |
| **2# sense** **5'- GGGAACAGAUUAGAAAUCUUCAGAG -3’** | | |  |  |
| **3# sense 5'- GACUAUAAUGCUGAGAACUGCUUCA -3’** | | |  |  |
|  | | |  |  |
| **LMNA siRNA sequence (human)** | | |  |  |
| **1# sense 5'- GCAGACCAUGAAGGAGGAATT -3’** | | |  |  |
| **2# sense 5’- GCUGCGCAACAAGUCCAAUTT -3’** | | |  |  |
| **3# sense 5’- CACCAAAGUUCACCCUGAATT -3’** | | |  |  |
|  | | |  |  |
